# Near Infrared Spectroscopy Calibration for Wood Chemistry: Which Chemometric Technique Is Best for Prediction and Interpretation?

**DOI:** 10.3390/s140813532

**Published:** 2014-07-25

**Authors:** Brian K. Via, Chengfeng Zhou, Gifty Acquah, Wei Jiang, Lori Eckhardt

**Affiliations:** 1 Forest Products Development Center, School of Forestry and Wildlife Sciences, Auburn University, 520 Devall Dr., Auburn, AL 36849, USA; E-Mails: czz0024@auburn.edu (C.Z.); gea0002@tigermail.auburn.edu (G.A.); weijiangqd@gmail.com (W.J.); 2 Center for Bioenergy and Bioproducts, Biosystems Engineering, Auburn University, 520 Devall Dr., Auburn, AL 36849, USA; 3 College of Textiles, Qingdao University, 308 Ningxia Road, Qingdao 266071, China; 4 Forest Health Dynamics Laboratory, School of Forestry and Wildlife Sciences, Auburn University, 602 Duncan Drive, Suite 3301. Auburn, AL 36849, USA; E-Mail: eckhalg@auburn.edu

**Keywords:** NIR, chemometric, PLS, PCR, regression, loading, coefficient, error, wood chemistry

## Abstract

This paper addresses the precision in factor loadings during partial least squares (PLS) and principal components regression (PCR) of wood chemistry content from near infrared reflectance (NIR) spectra. The precision of the loadings is considered important because these estimates are often utilized to interpret chemometric models or selection of meaningful wavenumbers. Standard laboratory chemistry methods were employed on a mixed genus/species hardwood sample set. PLS and PCR, before and after 1st derivative pretreatment, was utilized for model building and loadings investigation. As demonstrated by others, PLS was found to provide better predictive diagnostics. However, PCR exhibited a more precise estimate of loading peaks which makes PCR better for interpretation. Application of the 1st derivative appeared to assist in improving both PCR and PLS loading precision, but due to the small sample size, the two chemometric methods could not be compared statistically. This work is important because to date most research works have committed to PLS because it yields better predictive performance. But this research suggests there is a tradeoff between better prediction and model interpretation. Future work is needed to compare PLS and PCR for a suite of spectral pretreatment techniques.

## 1. Introduction

Near infrared spectroscopy (NIR) is becoming increasingly important for the rapid characterization of wood tissue chemistry. It is rapid and sometimes non-destructive if no grinding is required prior to analysis. In forests and materials derived thereof, NIR has been utilized to predict lignin [1], cellulose [2], hemicellulose [3], extractives [4], cellulose crystallinity [5], *p*-hydroxyphenyl (H)-, guaiacyl (G)-, and syringyl (S)-based lignin quality [6]. Secondary traits that depend on or correlate to the underlying wood chemistry have also been modeled with NIR spectroscopy including microfibril angle [7], tracheid morphology [8], mechanical properties [9], Kraft pulp yield [10], density [11], shrinkage behavior [12], moisture content [13], sapwood:heartwood ratio [14], and compression wood [15]. Assessment of these type of traits have been used to evaluate forest materials in genetic breeding trials [16,17], silviculture [18], forest products [19], pulp and paper [20], heat treatment [21], and bioenergy [22]. In most cases, the predictive capacity of the NIR model has been the focus of discussion with partial least squares (PLS) working better than principal components regression (PCR) for improved r^2^ and other predictive diagnostics. However, more and more scientists are using the coefficients/loadings within the models to interpret the relationship between wood chemistry functional groups and key traits including tensile strength [23,24], bending [11,25], calorific content [26], among others. But currently, it is unknown if PLS loading plots are statistically similar to that obtained from PCR. In the social sciences discipline, they have cautioned that PLS can be inferior for interpretation [27] while others have warned that shifts in loading location can occur in both PLS and PCR causing error during band assignment and consequent interpretation [28].

During prediction, investigators use these PLS or PCR loadings to assign specific wavenumbers to a chemical compound by assessment of the coefficients (loadings) of statistically significant principal components (PCs), but there may be random error associated with the estimation of these coefficients resulting in some level of uncertainty with either PCR or PLS. It is thought that these errors in PCR could be further inflated during PLS since the location of the “peaks” (coefficients of high and low local values at a given wavenumber) could shift when simultaneously adjusting the X and Y matrix for improved prediction. Any shift in these peaks would result in a wavenumber selection that would be slightly different than the real population value. It is thus important to investigate the precision and accuracy of the location of these peak loadings during modeling.

The first derivative has been shown to reduce the severity of the covariance of these adjacent wavelengths [29] in native spectra and it is hypothesized that such a pretreatment would improve the precision of the coefficients/loadings during modeling. But since the first derivative creates a new peak at the location where the slope was the maximum on the raw spectra, then the accuracy of these peaks will be compromised and thus application of the first derivative may improve the precision but lower the accuracy.

PCR and PLS regression are two multivariate techniques that are usually necessary to overcome the strong covariance in light absorbance between adjacent wavenumbers within a small region of the spectra which inflate the coefficients of the standard multiple linear regression equation [30]. The basic equations for PCR has been described elsewhere [31,32] and is a data reduction-multiple linear regression tool in which significant principal components (PC) are regressed against the dependent variable to construct calibration models of the chemical constituent. The coefficients (loadings) of a specific principal component are then used as weights to express the relative level of influence the original absorbance at that wavenumber has on the overall PC variance and consequently the chemical constituent associated with that PC. These coefficients are assumed to be continuous in nature and a smooth line is used to connect the coefficients resulting in an ability to identify local maximum and minimum loadings in the form of “peaks”. These coefficients are then used for interpretation, spectra reduction and remodeling, or in some cases even specific band assignment. The association of functional groups with specific wavenumbers can be made by referencing the literature [28] or regressing it against the chemical component of interest.

One potential weakness of PCR, at least for precision during prediction, is that the PC are developed only from the X data matrix and there is no consideration for the Y matrix until the PC are regressed against Y. The solution to this problem was the development of PLS regression in which the covariance between X and Y is taken into account during PC development [33]. This results in a slightly different data matrix that improves the covariance between the X and Y data matrix [33] and results in a higher r^2^ for the calibration model. But it is postulated that an improvement in covariance between the X and Y data matrix will result in a shift in the coefficient location (wavenumber) resulting in inflated error in “peak” location and consequent error during wavenumber selection, interpretation, or band assignment. Conversely, PCR may be a better tool for interpretation/explanation of the model [27] because PLS creates parameter estimates that maximize the covariance between the X and Y matrix. Thus PLS is more focused on prediction [27] while PCR may better preserve the original X-matrix structure resulting in better model interpretation.

As mentioned earlier, in the forestry and forest materials sector, research has increasing to interpret the coefficients relation to key functional groups and/or the underlying wood chemistry responsible for the response in the Y variable. Even more work has been done evaluating the predictive capacity of NIR. For example, NIR was used to quantify the patterns of extractives and klason lignin content both radially and longitudinally within 10 *Pinus palustris* trees [34]. The spectral measurements on these trees were obtained from solid wood surfaces and then related to the wood chemistry. But later it was found that there was supplementary error during prediction when solid wood was used because the tangential, radial, and longitudinal surfaces yields different absorbance patterns during spectra collection [35,36]. Likewise, the radial face was used during the prediction of lignin and monosaccharides which helped to control the predictive error [37]. These technical issues are important because it determines the precision and accuracy of the NIR model to characterize tree tissue chemistry which can impact wood quality based issues [38–41].

Grinding of plant tissues has proven useful during the reduction of prediction error while improving model robustness. For instance, solid wood was ground to 20, 40, and 80 mesh to see if model precision and consequent r^2^ could be improved [42]. It was found that predicted lignin content exhibited an r^2^ ≈ 0.6 when the spectra was acquired from the solid wood, increased to an r^2^ ≈ 0.9 at 20–40 mesh, and increased to an r^2^ = 0.96 – 0.99 for 80 mesh. Grinding was also recommended for *Pinus taeda* which improved predictions of whole tree properties [43]. For other plant materials such as ramie, grinding was also necessary to achieve stronger calibrations of lignin and cellulose [44]. But in all of these cases, the emphasis has been on increasing the predictive r^2^ or to reduce the predictive error of the Y matrix. To do this, investigators have often chosen PLS over PCR because many studies have demonstrated PLS to have higher predictive capacity. For illustration, lower predictive errors were achieved and with fewer factors when PLS was compared to PCR for the prediction of ash and char content [45]. When visible spectral data was applied to pulp samples, PLS also proved to be slightly more accurate once the optimal number of factors was determined [46]. Similar findings were established when ATR-FTIR was used to predict the delignification of lignin due to rotting fungi [47]. PLS was found to work better than PCR for both NIR and ATR-FTIR for the monitoring of the proximate analysis and heating value of torrefied switchgrass (*Panicum virgatum*), *Pinus taeda*, and *Liquidambar styraciflua* [31].

The primary objective of this paper was to investigate whether the PLS method introduces additional error in the loading plot, when compared to PCR, due to shifts in the loading peaks that might occur during the process of optimizing the covariance between the X and Y data matrix. As such, the alternative hypothesis (h_a_) for this experiment is that the loadings/coefficients in the PLS will decrease in precision and consequently increase in variance. To test this hypothesis, the location of the local peak loading, obtained through PLS and PCR coefficient plots, will be subtracted from the best representative band assignments as obtained from the literature:

(1) $$\text{C}\;-\;{\text{BA}}_{\text{L}}=\;\text{R}$$

where C represents wavenumber obtained through PLS or PCR analysis; BA_L_ is the best representative band assignment obtained from the literature; and R represents the Residual between C and BA_L_. Then the variance of the residuals will be further tested under the following hypothesis constructs:

(2) $${\text{H}}_{0}:\;{\sigma^{2}}_{\text{PLS}-\text{R}}={\sigma^{2}}_{\text{PCR}-\text{R}}$$

(3) $${\text{H}}_{\text{a}}:\;{\sigma^{2}}_{\text{PLS}-\text{R}}>{\sigma^{2}}_{\text{PCR}-\text{R}}$$

where σ^2^ represents the variance of R obtained from PLS or PCR models. Differences for variance between model loadings will be tested by the F-Test of R [30].

## 2. Experimental Section

### 2.1. Sample Preparation

All samples were collected from recently harvested hardwood trees. There were four different genera and 37 samples including four *Eucalyptus*, nine cotton wood, 12 aspen and 12 poplar. First, the wood samples were planed down to 3 mm thick wood chips and then stored for 2 weeks at 24 ± 1.5 °C and 45% ± 5% relative humidity. Two weeks was enough time to reach equilibrium with the environment; *i.e.*, the weight of the biomass no longer decreased with time. Then 50 g of air dried samples were ground to 40 mesh using a Willey mill and then 20 g of 40 mesh samples were further ground to 80 mesh. The 40 mesh samples were used for wood chemistry analysis (wood chemistry section) and the 80 mesh samples were used for FT-NIR spectra collection (FT-NIR acquisition).

### 2.2. Wood Chemistry

**Figure 1.**
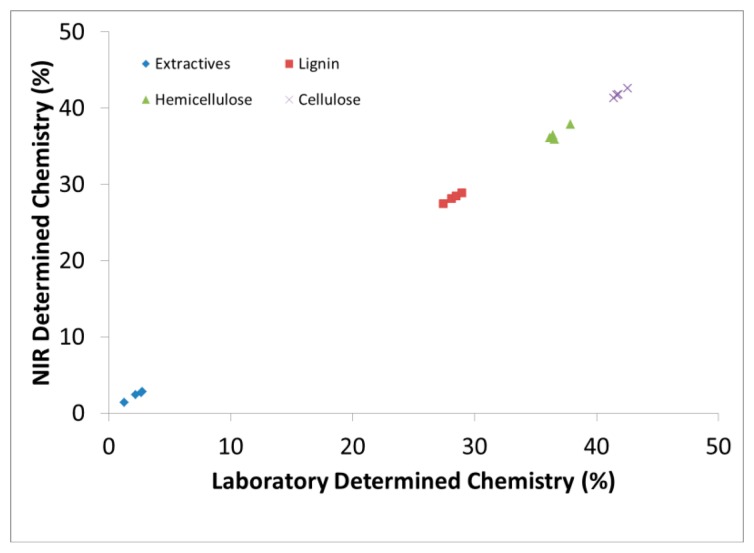
Chemical content (%, w/w) measured both in the laboratory and that predicted by NIR for validation samples.

The extractives, lignin and monosaccharide contents of 37 samples were measured following National Renewable Energy Laboratory (NREL) standards [48,49].The cellulose, hemicellulose and holocellulose contents were also measured by traditional wet chemistry analysis. As shown in Figure 1, 150 mL acetone was used to extract 5 g of sample for 6 h to get acetone based extractives. After extractives removal, the extractive free sample was separated into 2 batches.

Batch 1: A 72% (w/w) sulfuric acid treatment at 30 °C for 2 h was used to prehydrolyze the extractive free sample. The solution was then diluted to 4% sulfuric acid with distilled water, sealed in a bottle and placed in an autoclave for 1 h at 121 °C, and then the residual from the bottle was filtered and oven dried to measur lignin content. The extractives and lignin contents were measured by gravimetric analysis. To determine the monosaccharide composition, an HPLC (Shimadzu LC-20A), equipped with an Aminex 87 P column and differential refractive index detector and the sugar solution was analyzed. Holocellulose content (Holo-HPLC) was calculated as the sum of all the monosaccharides contents.
Batch 2: Delignification treatments were conducted to determine the holocellulose content. The delignification procedure was as follows. First, 2 g was weighed separately and placed into conical flasks (500 mL) with 320 mL of distilled water in each flask. Second, the flasks were placed into a water bath (75 °C) and the samples were placed into the flasks. Then 1 mL of acetic acid and 20 mL 15% (w/w) sodium chlorite were added into each flask on a 1-h cycle for 4 h. After 4 h, the residues were filtered with filter paper and then oven dried for 3 h to test the holocellulose content. Then, 1.5 g of oven dried holocellulose was placed into a 250 mL conical flask. One-hundred mL of 17.5% sodium hydroxide was stirred into the flask and the air was replaced with nitrogen and the flask was immediately sealed with aluminum foil. The flask was then placed in a water bath at 20 °C and stirred occasionally until the reaction was complete. The solution was then filtered through a pre-weighed filter paper and washed with 500 mL of distilled water. The sample was then oven dried at 105 °C for 12 h and weighed. The residue was determined as cellulose and the hemicellulose content was considered to be the difference in holocellulose and cellulose.

When conducting wet chemistry, all samples were air dried and tested for moisture content to calculate the dry weight of the original samples and such that moisture was not included as weight during gravimetric determination of the wood polymers. All experiments were performed in duplicate. All chemicals were purchased from VWR Company (Atlanta, GA, USA), and were analytically or chromatographically pure.

### 2.3. FT-NIR Acquisition

Samples were oven dried for 12 h and then placed into a dessicator to maintain near ovendry conditions but remove the effect of changing temperature on spectra fluctuation [50]. For each sample, the wood powder was placed on the FT-NIR machine to avoid packing and the reflectance spectra were collected on a window that was 8 mm in diameter. A PerkinElmer (Waltham, MA, USA) spectrum 400 FT-NIR spectrometer was utilized for spectra collection. The spectra covered the range of 10,000–4,000 cm^−1^ at a spectral resolution of 4 cm^−1^. Each spectrum was collected from an average of 32 scans and no zero filling. It should be noted that no smoothing was applied to the raw spectra because after 16 scans, there was no difference in the spectra before or after smoothing. Thirty-two scans were thus chosen for superior precision. Baseline analysis was also run on the raw spectra but there was no change in loading peaks so the raw spectra were analyzed with no pretreatment.

### 2.4. Chemometric Analysis

PCR and PLS modules in Spectrum Quant + software was used for model construction. Models were executed on the unprocessed spectra (raw) and first derivative (FD). The FD was computed prior to PCR or PLS modeling and was calculated with the Savitzky-Golay approach (2nd order polynomial with 25 points). Thirty-one samples were used to construct models and 6 samples were used for validation. Because of the small sample size, cross validation on all 37 samples (leave one out 37 times) was also ran to ensure similar results and insulate against one data point (out of five) biasing or inflating parameter estimates during validation. While the population for calibration and validation were randomly selected, the distribution of the data was checked to ensure a similar mean and range between the two populations. The predictive performance of the models in this paper was evaluated by several standards, including the coefficient of determination (r^2^), root mean square error of calibration (RMSEC), and root mean square error of prediction (RMSEP) [30]. The residual predictive deviation (RPD) was also measured to understand whether models could potentially be used in real measurement systems, screening, or just for interpretation purposes [42].

For PCR coefficient/loading plots, the most statistically significant PC to relate to the chemical constituent during multivariate modeling was utilized. The coefficients (*y*-axis) were connected via a smooth line in Origin software and then plotted against the wavenumbers (*x*-axis). For PLS, the regression coefficients plot was computed which represents the relationship between all of the absorbance (entire wavenumber range) and the specific chemical constituent of interest. The peak locations were then chosen and compared to wavenumbers chosen a-priori from the literature.

## 3. Results and Discussion

### 3.1. Predictive Diagnostics

Table 1 demonstrates the summary statistics for the best predictive models. In every case, PLS outperformed PCR in predictive diagnostics (not shown). Application of the 1st derivative resulted in better calibration models than when the raw spectrum was utilized. Preprocessing with the 1st derivative demonstrated better prediction based on the higher r^2^, a lower RMSEP, and a higher RPD. The sometimes drastic improvement in prediction with the 1st derivative was perhaps due to the presence of a baseline shift which can impact the computation of the 1st PC. These differences in model performance before and after derivative pretreatment was not expected. It was our pre-conjecture that grinding to a fine powder (80 mesh) would minimize any inherent solid wood density variations between samples which can cause baseline shifts in the spectra [42]. Similar improvements were observed during the prediction of wood cellulose crystallinity when the 1st derivative was applied and they also milled their samples [5]. Others have supported that baseline shifts and bias is often unavoidable in NIR spectra due to subtle differences in path length and differences in light scattering between samples [51]. It was also possible that some particles settled resulting in increased variation in bulk density although every effort was made to minimize this effect. It was also noticed that for smaller sample sizes such as in this and other studies from our laboratory [42], pretreatments were more necessary for calibration improvement than when larger data sets were employed [31].

Figure 1 demonstrates the capability to predict new samples based on calibration models and how one can simultaneously predict several wood chemical constituents from one spectral measurement. The predictive statistics in Table 1, however, were obtained through cross validation (leave one out method). We chose to use both methods to demonstrate the validity of the models but focused more on the cross validation method during model selection which has been shown to be better for small data sets [30].

### 3.2. Assessment of Loading Plots

**Figure 2.**
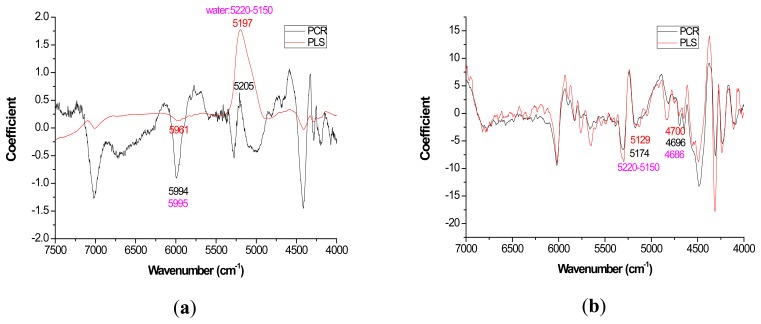
Coefficients by wavenumber for PCR and PLS for extractives prediction (**a**) when raw spectra was processed and (**b**) when a first derivative pretreatment was processed. PC number 9, 1, 5, and 3 were chosen for PCR-raw, PLS-raw, PCR-derivative, and PLS derivative respectively (α = 0.05).

For most loading plots, application of the 1st derivative resulted in more similar plots between PLS and PCR. For extractives prediction, the loading patterns were very dissimilar for the two modeling methods when the raw spectra were utilized (Figure 2).

The absolute magnitude of the coefficients was higher for PCR while PLS exhibited flatter plots with perhaps the only distinctive peak occurring at 5197 cm^−1^ (Figure 2a). However, when the first derivative was applied, there was no visual difference in coefficient intensity between PCR and PLS (Figure 2b).

**Figure 3.**
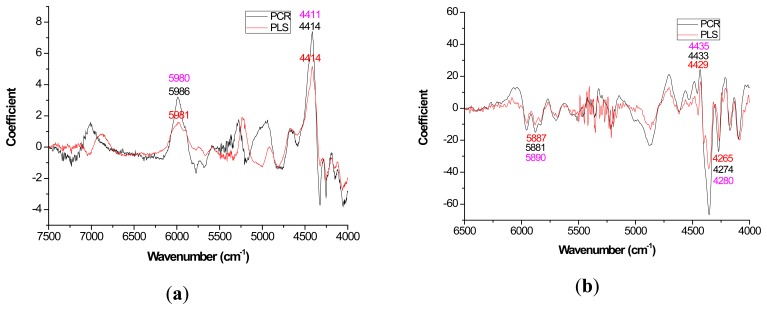
Coefficients by wavenumber for PCR and PLS for lignin prediction (**a**) when raw spectra was processed and (**b**) when a 1st derivative pretreatment was processed. PC number 9, 4, 5, and 3 were chosen for PCR-raw, PLS-raw, PCR-derivative, and PLS derivative respectively (α = 0.05).

**Figure 4.**
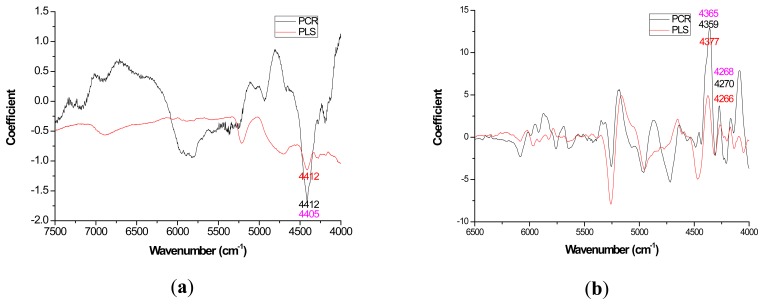
Coefficients by wavenumber for PCR and PLS for cellulose prediction (**a**) when raw spectra was processed and (**b**) when a 1st derivative pretreatment was processed. PC number 10, 8, 4, and 4 were chosen for PCR-raw, PLS-raw, PCR-derivative, and PLS derivative respectively (α = 0.05).

**Figure 5.**
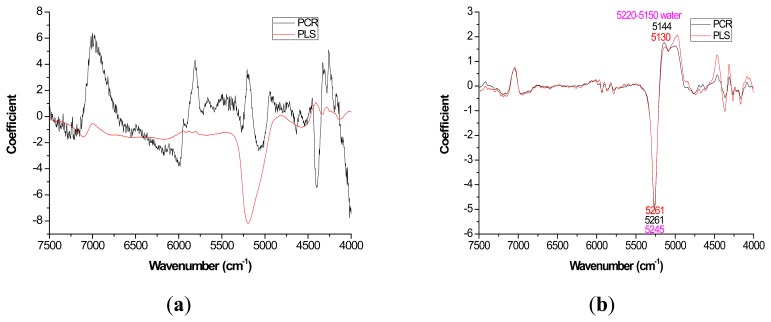
Coefficients by wavenumber for PCR and PLS for hemicellulose prediction (**a**) when raw spectra was processed and (**b**) when a 1st derivative pretreatment was processed. PC number 2, 1, 1, and 1 were chosen for PCR-raw, PLS-raw, PCR-derivative, and PLS derivative respectively (α = 0.05).

Mild improvements in PLS coefficient estimates (when compared to PCR) also occurred with the first derivative for lignin but once again PCR coefficients were slightly higher for the raw spectra based models (Figure 3a). For cellulose and hemicellulose, coefficient plots between the two methods became much more similar after first derivative application (Figures 4–5). In other words, the loading plots became more “parallel” or similar in pattern.

The general improvement in PLS coefficient plots with a 1st derivative pretreatment was probably attributable to the removal of the baseline shift that occurs due to physical rather than chemical features of the material and 25 point smoothing. For *Pinus palustris*, and *Pinus spp.*, it was demonstrated that an increase in solid wood density coincided with a linear increase in absorbance [35,52]. With PCR, the first PC will partition the variation due to the baseline shift such that better signals attributable to the underlying chemistry can be resolved through other PC [53].

With careful evaluation, it was also noticeable that there was sometimes a shift in the location of the peak coefficient when going from the native to 1st derivative based data sets. To illustrate, for the extractives models, the wavenumber at 5197–5205 cm^−1^ shifted to 5174 cm^−1^ when the 1st derivative pretreatment was used. This 20 to 30 cm^−1^ shift in absolute maximum coefficient could also be seen for lignin (4411 to 4435 cm^−1^) and hemicellulose (5225 to 5245 cm^−1^) (Figures 2 and 4). These errors will be quantified statistically later in the paper (Table 2).

For a given local region, this shift in location of the peak coefficient can be explained by the fact that the peak in the first derivative occurs at the same location as an inflection point location in the native spectra. A solution to this problem would be to take the 2nd derivative which will theoretically fall in the same location while simultaneously removing the baseline shift effect. However, with each derivative applied, the risk of lower signal to noise ratio increases which will have unknown effects on the prediction of the chemistry of future populations. This concept was demonstrated for blue stained tissue in which the confidence intervals for absorption were wildly inflated when transitioning from the 1st to 2nd derivative [54]. In that study, application of the 1st derivative maintained statistically similar confidence intervals as that obtained with the native spectra [55].

### 3.3. Interpretation of Significant Coefficients and Loadings Error Assessment

In this study, for the prediction of lignin, the O-H, C-O, C-H stretch and the aromatic skeletal vibrations were important loadings at 4401, 4411, and 4280 cm^−1^ respectively [28]. For extractives prediction, the C-O and O-H bond was important based on loadings at 6913 and 7092 cm^−1^ [28]. For cellulose prediction, the C-H and CH2 deformation were key functional groups that were important based on loadings at 6307, 5814, 4405 cm^−1^ [28]. Hemicellulose quantification yielded C-H and C=O bond based on loadings at 7410, 6003, 5236, and 4686 cm^−1^.

**Figure 6.**
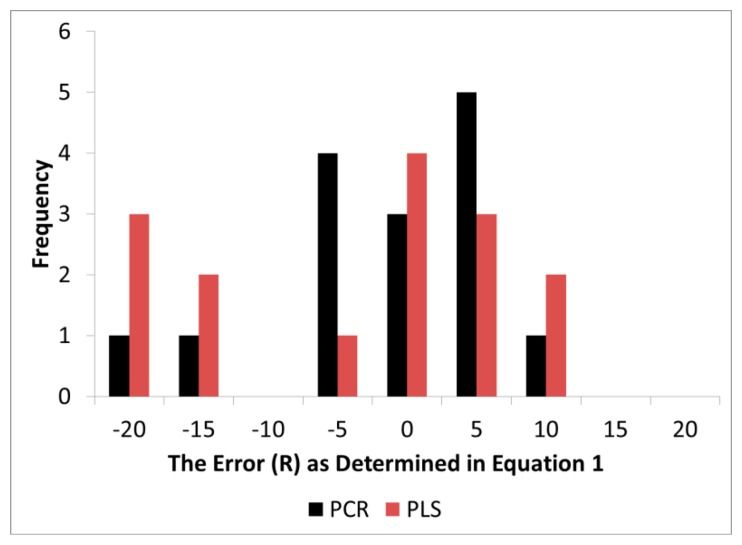
Frequency of R from PLS and PCR loadings of wood chemistry models.

It should be noted that band assignments given above were those standard to the literature (BA_L_) and were chosen *a priori* while the loadings in the models, as anticipated, exhibited some level of error around BA_L_. The distribution of error (R) for both PCR and PLS exhibited a skewed pattern while PCR exhibited a distribution closer to normality (Figure 6). Visually, the error appeared to be slightly biased to one side of zero, but when a confidence interval test was performed for both PLS and PCR (α = 0.05), both overlapped with zero (Table 2). Thus statistically, both methods were still accurate for proper wavenumber selection and assignment. Nevertheless, the R (distribution of error) in PLS was higher in variance and this made it more difficult to determine if the mean differed from zero through confidence interval testing for the number of degrees of freedom available. As Figure 6 demonstrates, it is quite possible that PLS did introduce bias in loading location.

The precision of the location of the peak loading was tested through hypothesis testing. The alternative hypothesis H_0_ was developed because it was believed that PLS will compromise loadings estimates in order to maximize X and Y matrix covariance. An F-Test revealed that the variance in R for PLS was statistically greater than PCR. This means that while PLS exhibited better prediction of wood chemistry, the error (R) in the loadings estimates increased making wavenumber selection through modeling less certain with PLS.

The precision of PCR for identification of peak location has been adjacently investigated and explained by similar research in the field of 2D correlation spectroscopy in which a perturbation was added to improve the precision of band identification during shift [56]. Two-dimensional correlation spectroscopy and waterfall plots was explored to decipher subtle peak shifts which was a challenge due to overlapping wavenumbers within the local IR region. They explored principal components analysis (PCA) as a supplementary method for monitoring band shift and they were surprised to find this analytical tool to be very sensitive to true maximum peak shift. Their research findings perhaps suggests that the peak variance found in our PCR analysis may be more inherent to sample to sample variation while we think the additional peak variance introduced during PLS analysis (Table 2) for this study was the result of X-matrix modification during X-Y covariance optimization.

Similar to Ryu *et al.* [56] and this study, another similar finding was present for the analysis of NIR spectra which was acquired from longleaf pine (*Pinus palustris*) [29]. When the 1st derivative pretreatment was applied and then spectra separated based on stiffness and strength perturbations, lignin and cellulose associated wavelengths were easily separated while hemicellulose was not discernable. But when PC loadings were investigated, they witnessed a significant peak at 2330 nm attributable to the CH stretch in hemicellulose. In our study, there was not a statistical difference between the error (R) in PCR and PLS after application of the 1st derivative (α = 0.05). It is thus thought that the 1st derivative may be a tool to improve loading plot precision; however, most of the degrees of freedom were necessary for testing the original hypothesis that PLS and PCR (in general) differed in error. We thus recommend a separate study in the future to better quantify the improvement in precision with a derivative application. Indeed PCA has recently been shown to be more sensitive to spectra variance for samples with complex reactions or for wood chemistry that possesses similar functional groups. Perhaps that helps to explain it utility as a superior interpretation tool during chemometric modeling such as that employed in this study.

### 3.4. Closing Observations

In closing, the data analysis for this study was the first of its kind, in part, because it took several models (raw and 1st derivative) and multiple wood chemistry traits combined to yield enough degrees of freedom to test for significant differences in targeted loadings between PLS and PCR (Table 2). Unfortunately, the sample size was too low to further test for significant differences in R for native *versus* 1st derivative spectra. Since the loadings (Figures 2–5) appeared more stable for 1st derivative based models, future work is necessary to determine if R decreases for PLS after pretreating with the 1st derivative and this work should be compared to PCR. Other pretreatments or even 2D correlation may also be useful for reduction in R and should also be tested for in future studies.

## 4. Conclusions

The main purpose of this study was to assess the performance of PLS and PCR for interpretation purposes. In order to do that, we had to quantify the potential error in loading plots and in particular any deviation in the location of the “peaks” from the true population value. It was found that PLS did a better job during prediction while PCR exhibited better precision in identifying the correct loading position and consequently PCR would be better for model interpretation, wavenumber selection, or similar activities. Application of the 1st derivative appeared promising in that the shapes between PCR and PLS loading plots became more similar or “parallel”. But future research is necessary to better understand if the 1st derivative or any other pretreatment can yield PLS loading plots with better precision than what was obtained in this study.

This work is important because it suggests that what is best for prediction is not best for model interpretation. Currently, most papers focus on PLS because it does a better job at prediction. But by nature, when the same investigators transition to model interpretation, they may be biased toward PLS because of its superior predictive nature and their prior use of the model. We prescribe that chemometricians consider PCR for their toolbox when performing model interpretation.

## Figures and Tables

**Table 1.** Calibration and predictive results of NIR based multivariate (PLS) models.

**Chemistry**	**Raw Spectra**			**First Derivative**		
	
	**r^2^**	**RMSEP**	**RPD**	**r^2^**	**RMSEP**	**RPD**
Extractives	40.7	1.18	1.21	85.0	0.98	1.45
Lignin	82.6	1.35	1.78	90.4	1.12	2.15
Cellulose	37.6	2.10	1.00	81.0	1.03	2.04
Hemicellulose	41.9	3.17	1.23	93.5	1.43	2.72

**Table 2.** Hypothesis testing of Equations (2) and (3) through the F-Test. * means the F-Test was significant with 95% confidence.

	**PCR**	**PLS**
Mean R	−3.4	−9.4
Variance	189	700
Standard deviation	13.7	26.5
95% CI	−3.4 ± 7.6	−9.4 ± 14.7
Observations	15	15
Degrees of freedom	14	14
F	0.27	
P (F < f) one tail	0.0099 *	
